# Modified Child Dental Anxiety Scale-Faces (MCDASf): validity and reliability of the Brazilian version

**DOI:** 10.1590/1807-3107bor-2025.vol39.104

**Published:** 2025-10-10

**Authors:** Marília Leão GOETTEMS, Fernanda Vieira ALMEIDA, Giulia Tarquinio DEMARCO, Marina Sousa AZEVEDO, Vanessa Polina Pereira da COSTA, Taís de Souza BARBOSA

**Affiliations:** (a) Universidade Federal de Pelotas – UFPel, Postgraduate Program in Dentistry, Department of Social and Preventive Dentistry, Pelotas, RS, Brazil.; (b) Universidade Federal de Pelotas – UFPel, Postgraduate Program in Dentistry, Pelotas, RS, Brazil.; (c) Universidade Estadual Paulista – Unesp, Institute of Science and Technology, Department of social and Pediatric Dentistry, São José dos Campos, SP, Brazil.

**Keywords:** Dental Anxiety, Pediatric Dentistry, Validation Study, Surveys and Questionnaires

## Abstract

Specific measures for assessing dental anxiety and fear are crucial in pediatric dentistry for their management. This cross-sectional study aimed to test the validity and reliability of the Brazilian Portuguese version of the Modified Child Dental Anxiety Scale – Faces (MCDASf). A total of 189 children enrolled in public schools in Pelotas, Southern Brazil, and their parents were included. The children completed an interview that included the MCDASf, Children’s Fear Survey Schedule-Dental Subscale (CFSS-DS), Dental Anxiety Question (DAQ), and self-reported oral health-related outcomes. The parents provided sociodemographic data and information on dental visits and pain. Descriptive statistics, comparison and correlation tests, and psychometric analyses were conducted. The MCDASf scores were positively correlated with the CFSS-DS scores (rho = 0.60; p < 0.001). Children reporting dental anxiety (DAQ) had higher MCDASf scores. Exploratory factor analysis (EFA) was conducted on MCDASf items. The factor model was supported by Bartlett’s test of sphericity (p < 0.001) and the Kaiser-Meyer-Olkin (KMO) measure (0.782). The loadings indicated that the two factors explained 18.9% and 13.9% of the variance. Factor 1 included items 1–3, 5, 7, and 8, whereas Factor 2 included items 4 and 6. The MCDASf showed satisfactory internal consistency: Cronbach’s α = 0.73 and McDonald’s ω = 0.82 for the total scale, with α and ω > 0.66 for both factors. Coefficients ≥ 0.70 are considered acceptable. This study provided psychometric evidence supporting the Brazilian MCDASf (B-MCDASf) as a reliable self-report tool for assessing dental anxiety in children in both clinical and research settings.

## Introduction

Dental fear is defined as a normal emotional reaction to one or more specific threatening stimuli in the dental environment, whereas dental anxiety denotes a state of apprehension that something dreadful related to dental treatment can happen and is coupled with a sense of losing control.^1^ In literature, the terms dental anxiety” and “dental fear” are often used interchangeably. In the context of dental treatment, distinguishing between fear and anxiety during clinical situations is difficult.^2^ Treating patients with dental fears/anxiety (DFA) presents various challenges for dental professionals. Thus, assessing DFA is an important step in its management.^2^.

Several assessment tools are available for evaluating the DFA in both research and clinical practice. Different measures operationalize the construct of childhood DFA differently.^3^ Among the self-reporting measures that are the main methods for DFA assessment, some of the most frequently used are the Venham Picture Test (VPT)^4^ and the Facial Image Scale (FIS),^5^ which assess state anxiety (how a child is feeling at the moment), and the Children’s Fear Survey Schedule-Dental Subscale (CFSS-DS)^6^ and the Modified Child Dental Anxiety Scale (MCDAS),^7^ which assess trait anxiety (individual proneness to DFA).

The MCDAS, developed in English, is a short self-report tool for children, consisting of 8 items that assess the severity of DFA in relation to typical dental situations. The MCDAS has demonstrated good internal consistency and validity.^7-9^ To improve the MCDAS response format, Howard and Freeman (2007)^10^ proposed the faces version (MCDASf), which associates a facial drawing with each of the five response options. This modification has been proven to be a valid approach to assess dental anxiety in the pediatric population.^10^ The MCDASf adapted and tested in various languages and cultures^11-13^ has consistently demonstrated valid and reliable results. However, the MCDASf has not been validated in Brazilian Portuguese, necessitating additional studies to confirm its applicability in Brazil. Cultural validation is essential to ensure that tools like the MCDASf accurately capture the nuances of dental anxiety in diverse populations, thereby enhancing their reliability and relevance. Cultural factors shape the expression and perception of dental anxiety, with some cultures encouraging children to suppress fear, potentially leading to under-reporting, whereas others may tolerate or even encourage the open expression of fear, resulting in more visible anxiety.^14^ These cultural differences emphasize the need for culturally adapted instruments to ensure validity across various populations. The Brazilian Portuguese version of the MCDASf (B-MCDASf) has been developed and found to be well understood and culturally appropriate;^15^ however, its validity and reliability still require testing. This study aimed to evaluate these aspects of B-MCDASf.

## Methods

This study was reported in accordance with Strengthening the Reporting of Observational Studies in Epidemiology (STROBE) guidelines.^16^ All steps were conducted in accordance with the Declaration of Helsinki, and the study protocol was approved by the local Human Research Ethics Committee under protocol number 5.494.403. Reliability and validity assessments were performed following the COnsensus-based Standards for the selection of the health Measurement Instruments (COSMIN) checklist.^17^

### Study design, settings and participants

This cross-sectional study was conducted between October 2022 and June 2023 and included children aged 5–12 years enrolled in two public schools in Pelotas, a city in southern Brazil. Schools were randomly selected from different neighborhoods in the city of Pelotas. The study was conducted in school settings with children, and a questionnaire was sent to parents through the school. Prior to data collection, the parents signed a consent form containing the details of the study. All children were also asked if they would like to participate and were required to sign an assessment form upon agreeing. Children with mental or physical disabilities that hindered their understanding of the instructions, and whose parents did not provide consent were excluded.

The sample size was calculated based on a study on the translation and cultural adaptation of the MCDASf into Brazilian Portuguese.^15^ Considering a mean (SD) of 17.6 (5.6), a 5% margin of error, a confidence level of 95%, and 80% power, the required sample size was determined to be 156 children.

### Data collection

The children were interviewed in a separate room by trained and calibrated undergraduate dental students on the school premises, and a questionnaire was sent to parents by the school. Before data collection, a pilot study was performed with 10 participants who were not included in the study to test the methodological approach and the respondents’ acceptance and compliance with the materials. Minor adjustments were accordingly made.

### Measures

The MCDASf^15^ assesses dental anxiety through eight questions related to various dental situations: ‘having your teeth looked at,’ ‘going to the dentist,’ ‘having your teeth scraped and polished,’ ‘having a mixture of gas and air to help you feel comfortable for treatment but not put you to sleep,’ ‘having a filling,’ ‘being put to sleep for treatment,’ ‘having a tooth taken out,’ and ‘having an injection in the gum.’ Responses are recorded on a five-point Likert scale, ranging from ‘relaxed/not worried’ (scoring 1) to ‘very worried’ (scoring 5). The scale is accompanied by five facial drawings that visually represent affective states from ‘extremely positive’ to ‘extremely negative.’ Children were asked to select the image that best corresponded to how they felt about each of the eight questions. The total scores range from 8 to 40; higher scores indicating greater levels of dental anxiety.

The Dental Anxiety Question (DAQ),^18^ a single-item scale translated into Brazilian Portuguese, was used to assess self-reported dental anxiety: “Are you afraid of going to the dentist”?. Responses are: “No,” “Yes, a little,” “Yes, quite,” or “Yes, very.” This variable was dichotomized into two categories: with anxiety (responses: “Yes, a little,” “Yes, quite” or “Yes, very”) and without anxiety (response: “No”).

Children’s dental anxiety was assessed using the modified Venham Picture Test (VPT).^4^ This tool uses sex-specific drawings of human figures printed on A4-sized colored paper to depict different emotional states. Children were asked to point to the figure that matched their feelings. The total score for all figure pairs ranged from zero to eight: zero, no anxiety; one to three low anxiety; four to six, medium anxiety; and seven to eight, high anxiety.

Children’s dental fear was also assessed using the Children’s Fear Survey Schedule-Dental Subscale (CFSS-DS). The CFSS-DS is a well-known questionnaire that assesses children’s dental fear^6^ using 15 items that refer to the clinical and psychological aspects of dental care.^6,19^

Sociodemographic variables assessed included parental education level (< 11 or ≥ 11 years), and monthly household income (up to 1 Brazilian Minimum Wage (BMW), up to 2 BMW or ≥ 2 BMW). The legal guardians answered whether the child had dental pain in the last 6 months (yes/no), whether they had visited a dentist (yes/no), and the reason for the visit (routine/preventive checkup, pain-related, or non-pain-related).

Children’s oral health perception was assessed through two questions: “When you think about your teeth or mouth, do you think they are: positive or negative, and “How much do your teeth or mouth bother you in your daily life? Not at all, somewhat, or very much.

### Statistical analysis

The statistical analysis was performed using the JAMOVI 2.3.17 statistical software, considering a significance level of α = 0.05. The Shapiro-Wilk test indicated a non-normal distribution of the data; therefore, non-parametric tests were used. Construct validity was determined by the relationship between the MCDASf values and the VPT and CFSS-DS scores using the Spearman’s correlation test. Criterion validity was assessed using the Mann-Whitney U test to compare the MCDASf means according to the DAQ response. Discriminant validity considered the MCDASf scores among the categories of the variables: sex, age, caregiver’s education level, dentist visits, pain in the last 6 months, income, child’s perception of their teeth, and whether the teeth bothered the child. The association of these independent variables with MCDASf as the dependent variable (> median 18) was tested for predictive validity using a multiple logistic regression model. Reliability was investigated using two measures of internal consistency: Cronbach’s alpha (α) and McDonald’s omega (ω). For both coefficients, values ≥ 0.70 were considered adequate.^20,21^

Exploratory factor analysis (EFA) was performed on a set of MCDASf items. The suitability of the data was tested using the Kaiser-Meyer-Olkin (KMO) measure of sampling adequacy, along with the Bartlett’s test of sphericity (KMO ≥ 0.5 and p < 0.001 show the adequacy of the data for use in the EFA). Initially, a 1-factor solution was explored that ranked the items according to their impact on the total variance of the scale. Factors were extracted using parallel analysis with promax rotation. Factor loadings < 0.4 indicate a low item impact on the instrument validity.^22^

## Results

The sample consisted of 189 children (mean age: 9.0 ± 1.9), 50.3% of whom were girls. There were no dropouts, as all questionnaires from parents who agreed to participate were evaluated and their respective children were included in the analyzed sample.

The mean total MCDASf score was 18.2 (5.7), with no difference between sex and age groups. There were also no significant differences between each item and sex or age groups (5–8 and 9–13 years) (data not shown).

The construct validity of the MCDASf was assessed by examining its correlation with the VPT and the CFSS-DS. A weak and non-significant correlation was observed between MCDASf and VPT scores (rho = 0.12; p = 0.07). In contrast, a moderate correlation was found between the MCDASf and CFSS-DS scores (rho = 0.60; p < 0.001).

Criterion validity was assessed by comparing the MCDASf scores according to the DAQ. Children who reported no dental anxiety on the DAQ had a lower mean MCDASf score (16.5; SD = 5.2) than those who reported some anxiety level (21.2; SD = 5.2) (p < 0.0001).

The discriminant validity is shown in Table 1. MCDASf scores differed significantly between children with and without dental experience. Children who had not visited a dentist had higher MCDASf scores than those who had (19.8 vs. 17.6; p = 0.017), indicating significant discriminant validity in relation to dental experience. No significant differences were observed in the other variables.

**Table 1:** Discriminant validity: MCDASf score according to sociodemographic characteristics, dental history and oral health perception (n = 189)

Variables	n (%)	Mean (SD)	p-value
Sex			
Male	94 (49.7)	18.1 (5.6)	0.498*
Female	95 (50.3)	18.3 (5.7)	
Age (years)			
5–8	80 (42.3)	18.3 (5.5)	0.610*
9–13	109 (57.7)	18.1 (5.8)	
Guardian’s level of education (YEARS) (n = 187)			
< 11	123 (65.1)	18.1 (5.5)	0.768*
≥11	64 (33.9)	18.4 (5.9)	
Visited a dentist			
No	53 (28.0)	19.8 (5.7)	0.017*
Yes	136 (72.0)	17.6 (5.5)	
Dental pain			
No	141 (74.6)	18.4 (5.8)	0.413*
Yes	48 (25.4)	17.7 (5.4)	
Family income (BMW) (n = 176)			
> 1	62 (35.2)	18.5 (5.7)	0.763**
> 2	62 (35.2)	17.7 (5.5)	
≥ 3	52 (29.5)	18.3 (5.8)	
Oral health perception			
Positive	72 (38.1)	18.5 (5.5)	0.417*
Negative	117 (61.9)	18.0 (5.7)	
Teeth bother the child			
Not at all	116 (61.4)	18.0 (5.4)	0.535*
Somewhat/Very much	73 (38.6)	18.6 (6.0)	

MCDASf: Modified Child Dental Anxiety Scale; SD: standard deviation; BMW: Brazilian minimum wage. *Mann-Whitney; **Kruskall-Wallis.

Predictive validity revealed that sex was a significant predictor of dental anxiety, with girls being nearly twice as likely to have higher MCDASf scores (OR = 1.91, p = 0.044). As per the multiple logistic regression model, approximately 4% of the variability in MCDASf scores above the median was explained by sex (Table 2).

**Table 2:** Predictive validity: multiple logistic regression considering the total MCDASf score > median as the dependent variable (n = 173)

Independent variable	Category	OR	95%CI	p-value	VIF	Tolerance
Intercept		1.58	0.57-4.42	0.374	-	-
Sex	Female	1.91	1.01-3.58	0.044	1.03	0.971
Age (years)	9–13	0.81	0.41-1.61	0.551	1.20	0.831
Guardian’s level of schooling	< 11 years	0.77	0.38-1.53	0.458	1.13	0.887
Dental pain	Yes	0.91	0.42-1.94	0.806	1.11	0.902
Visited dentist	No	0.57	0.27-1.18	0.131	1.10	0.907
Family income	Up to 1 BMW	0.86	0.44-1.68	0.669	1.06	0.946
Oral health perception	Negative	0.80	0.39-1.62	0.541	1.21	0.825
Teeth Bother the Child	Somewhat/Very much	1.10	0.57-2.15	0.764	1.08	0.929

Table 3 presents the factor structure of MCDASf obtained from the EFA. The appropriateness of this factor model was evaluated using Bartlett’s test of sphericity (p < 0.001) and the KMO measure of sampling adequacy (0.782). The estimated loadings indicate that MCDASf includes two factors that explain 18.9% and 13.9% of the total variance. Factor 1 combined six of the 8 items (1–3, 5, 7, and 8), whereas Factor 2 combined items 4 and 6.

**Table 3:** Factor loading from the Exploratory Factor Analysis, by the MCDASf items (n = 189).

MCDASf	How do you feel about…	Factor	
		1	2
(KMO = 0.782)	1. Going to the dentist generally?	0.439	
	2. Having your teeth looked at?	0.450	
	3. Having your teeth scraped and polished?	0.519	
	4. Having an injection in the gum?		0.718
	5. Having a filling?	0.602	
	6. Having a tooth taken out?		0.734
	7. Being put to sleep to have treatment?	0.433	
	8. Having a mixture of gas and air that will help you feel comfortable for treatment but will not put you to sleep?	0.519	
Total variance explained (%)		18.9	13.9

MCDASf: Modified Child Dental Anxiety Scale; KMO: Kaiser-Meyer-Olkin scale. Extraction method: Parallel analysis. Rotation method: Promax.

Table 4 presents the reliability of the results. A floor effect was observed in 3.2% of the samples, and no ceiling effect was observed. Reliability of the MCDASf was confirmed, with a Cronbach’s α of 0.73 and McDonald’s ω of 0.82, indicating substantial to excellent internal consistency. The values for these factors showed moderate internal consistency.

**Table 4:** MCDASf reliability: mean total score and by item, floor and ceiling effects and internal consistency (n = 189).

MCDASf reliability	Mean (SD)	Floor effect^a^	Ceiling effect^b^	Cronbach’s α (if item is deleted)	McDonald’s (ω )
Score total	18.2 (5.7)	6 (3.2)	0 (0.0)	0.73	0.82
Factor 1	11.4 (4.1)	27 (14.3)	0 (0.0)	0.66	0.67
1. Going to the dentist generally?	1.8 (0.9)	91 (48.1)	4 (2.1)	0.71	0.82
2. Having your teeth looked at?	1.9 (1.0)	91 (48.1)	6 (3.1)	0.70	0.81
3. Having your teeth scraped and polished?	1.5 (0.9)	126 (66.7)	1 (0.5)	0.71	0.82
5. Having a filling?	2.3 (1.4)	75 (39.7)	19 (10.1)	0.69	0.81
7. Being put to sleep to have treatment?	2.2 (1.4)	86 (45.5)	22 (11.6)	0.70	0.82
8. Having a mixture of gas and air that will help you feel comfortable for treatment but will not put you to sleep?	1.7 (1.0)	109 (57.7)	4 (2.1)	0.71	0.72
Factor 2	6.8 (2.5)	12 (6.3)	33 (17.5)	0.67	0.67
4. Having an injection in the gum?	3.3 (1.4)	25 (13.2)	54 (28.6)	0.69	0.80
6. Having a tooth taken out?	3.4 (1.4)	31 (16.4)	60 (31.7)	0.68	0.80

^a^n (%) of respondents with lower score (8); ^b^ n (%) of respondents with maximum score (40); MCDASf: Modified Child Dental Anxiety Scale; SD: standard deviation.

## Discussion

Dental care is complex, and the DFA can vary according to different situations.^2^ To assess children’s perceptions of dental situations, the MCDAS was developed based on the four dental scenarios of the Corah Dental Anxiety Scale (DAS) originally created for adults.^23,24^ The MCDASf consists of eight questions that evaluate DFA in relation to different dental situations but uses a simpler scale, as the numerical format proved challenging for children to comprehend. The present study suggests that this tool can be reliably used in Brazil to assess DFA in children, as it has demonstrated reliability and construct validity.

The mean MCDASf score in the present study was 18.2, similar to the findings of Javadinejad et al.^11^ and Honkala et al.^13^ Both studies included participants within the same age range, enhancing the comparability of the results across different contexts. Javadinejad et al. conducted their research in Iran, whereas Honkala et al. focused on Finland. The consistency in MCDASf scores across different geographical locations suggests that dental anxiety among children may possess universal characteristics despite cultural and environmental differences. However, the prevalence of dental fear has not been investigated at the national level in many countries, and individual studies may not have accurately captured this prevalence.^25^

The construct and criterion validity were assessed using well-established scales for measuring DFA in children. Significant correlations were found between the CFSS-DS and MCDASf, but not with the VPT. This can be justified because both the CFSS-DS and the MCDASf are considered trait measures, implying that they evaluate a constant background level of anxiety about dental treatment, with multiple items related to the construct of DFA.^26^ By contrast, the VPT is a state anxiety measure that captures a child’s immediate emotional response. Criterion validity was evaluated by comparing the Brazilian version of the MCDASf to the DAQ. Children who reported DFA had higher MCDASf scores.

To our knowledge, the EFA of MCDASf has been reported in three studies.^10,11,27^ In the original version of the MCDASf, a two-factor structure named “Examination” and “Treatment” that explained 55% of the variance was reported.^10^ Differently, in the Turkish version, a three-factor structure named “Dental sedation,” “General dental practice,” and “Dental examination” explained 61.75% of the variance. In the present study, a four-factor structure (“Invasive dental treatment,” “Dental examination,” “Non-invasive dental treatment,” and “Dental sedation”) explaining 47.6% of the variance^27^ was defined. In this study, 32.8% of the variance in the data was explained by two factors (1: Non-invasive dental treatment and 2: Invasive dental treatment). All factor loadings were acceptable (> 0.40)^20^ considering the sample size needed for significance (n = 150-200, 0.45-0.40) .^28^ Furthermore, as the cross-loading item is an item that loads at 0.32 or higher on two or more factors,^29^ the present findings must be interpreted with caution considering the values of the overall model fit (X^2^= 15.8; df = 13; p = 0.259).

In Factor 1, the loadings ranged from 0.43 to 0.60, with the greater values for items 3 (0.51, ‘*Having your teeth scraped and polished*’), 8 (0.51, ‘*Having a mixture of gas and air that will help you feel comfortable for treatment but will not put you to sleep?*’), and 5 (0.60, ‘*Having a filling?*’) in Factor 1. For Factor 2, values higher than 0.71 were observed for the two items related to invasive dental treatment. The factor structure was similar to that of the above-mentioned studies, and the differences could be explained by linguistic details and cultural particularities.^30,31^ Moreover, a lower proportion of variance in the present study than in previous studies^10,27^ can be explained by the multifactorial structure of the MCDASf.

Reliability was measured by internal consistency, with McDonald’s ω considered a more reliable estimator than Cronbach’s α, as it does not assume essential tau-equivalence.^32^ Both coefficients reached satisfactory levels for the total scale (> 0.70),^20,21^ indicating that the items were strongly related and measured the same construct effectively. For Factors, the coefficients ranged from 0.66 to 0.67; values higher than 0.60 were considered acceptable for a scale with a reduced number of items.^33^ The ceiling effect (percentage of individuals with the highest possible score) was above the acceptable 15% value for factor 2. Considering that these items are related to aversive dental procedures (4-dental anesthesia; 6-tooth extraction), a higher score (‘very worried, scoring 5) could be expected even in a sample of scholars.

Girls were found to have approximately twice the likelihood of higher dental anxiety scores, which is consistent with previous studies that showed that girls tend to report more anxiety than boys.^1,30^ However, in the predictive validity analysis, no differences were observed with respect to socioeconomic factors, oral health perception, or dental history.

This study has strengths that should be highlighted. Although a convenience sample was used, the required sample size was calculated and sample power was determined, lending credibility to the results. The study was also conducted in two different schools, allowing the participation of children who might have avoided dental visits due to DFA. As a limitation, only public schools were included, limiting external validity. Therefore, future studies should include more representative samples.

Dental anxiety is common among children and has significant clinical and psychosocial effects. Validated tools are essential to provide information on the prevalence and severity of DFA.^11^ In clinical settings, self-reporting instruments, such as the MCDAf, provide a valuable opportunity for empathetic conversations with young patients about their feelings towards dental treatment. This helps validate their feelings and allows treatment plans to be tailored to their specific anxieties and fears. Although self-reported DFA measures are often used in epidemiological surveys and research studies, it has been noted that dental professionals may not routinely incorporate formal DFA assessments in clinical practice. The MCDASf is a simple, quick, and effective self-report measure of trait anxiety that can be easily applied in both clinical practice and epidemiological studies. The Brazilian version of the MCDASf has been proven to be a valid and useful tool for assessing DFA in children.

## Conclusion

This study provided psychometric evidence to support the Brazilian version of the MCDASf as a reliable self-report tool that can be used in both clinical practice and research involving children.

MCDASf: Modified Child Dental Anxiety Scale; BMW: Brazilian minimum wage; OR: odds ratio; VIF: variance inflation factor; CI: confidence interval; R^2^_N_ = 0.071

## Data Availability

The datasets generated during and/or analyzed during the current study are available from the corresponding author on reasonable request.
